# 

**Published:** 1914-04-11

**Authors:** 


### Appointments.

Dr. J. F. Powell, second assistant medical officer, has been promoted by the Metropolitan Asylums Board to be a senior assistant medical officer in their mental hospitals service.

Mr. A. McNab, M.B., F.R.C.S., has been reappointed by the Metropolitan Asylums Board for another year as consultant in connection with the treatment of ophthalmia cases in mental hospitals at an inclusive fee of five guineas per visit, the maximum number of his visits in respect to all the institutions to be twenty.

Mr. David W. John, M.R.C.S., L.R.C.P. (Cantab.), has been appointed house physician to the Cardiff Infirmary following his appointment as house surgeon to the Gravesend Hospital.

The Metropolitan Asylums Board have renewed the appointment of Dr. H. G. Critchley as assistant physician for skin diseases (*x*-ray work). Dr. Critchley has hitherto been styled assistant dermatologist for *x*-ray work, but the title of dermatologist having been altered to that of consulting physician for skin diseases, a corresponding change has been made in the designation of Dr. Critchley.

The undermentioned have been appointed by the Metropolitan Asylums Board junior assistant medical officers in the infectious hospitals service :—Mr. G. Cochrane, M.A., M.B., Ch.B. (Glas.); Mr. F. Coplans, M.R.C.S., L.R.C.P. (Lond.), M.B. (Lond.); Mr. J. B. Low, M.B., Ch.B. (Edin.); Mr. K. A. Maclean, M.B., Ch.B. (Edin.); and Mr. G. H. Stevenson, M.B., Ch.B. (Edin.), D.P.H.

[For a note on the new Assistant Medical Officers' Association see page 32.]

THE

### Doctors' Wives Association.

(*See page* 36.)

### COUPON.

I hereby certify that I am a doctor's wife, and I beg to send you my name and address as one who wishes to become a member of the proposed Doctors' Wives Association. I hereby agree to qualify as a member by paying the subscription of 7*s.* 6*d.* per annum on or before May 1, 1914, such subscription entitling me each year to a free copy of The Hospital newspaper delivered weekly, with the Doctors' Wives section, and to include my annual subscription to the Association.

This agreement undertaking to subscribe on my part is subject to at least 1,000 doctors' wives conditionally joining the proposed Association by signing and returning to "Co-operation" as below by April 18 next a similar coupon to that to which I hereby attach my signature.

(Signed) ........................................

(Wife of) * ........................................

(Address) ........................................

........................................

(Date) ........................ ........................

*Note.*—When filled in and signed cut out this coupon, place it in an envelope and address and post it to :

Co-Operation,
c/o The Scientific Press,
28 & 29 Southampton Street, Strand,
London, W.C.

* Here insert full name and qualifications.

RATES OF SUBSCRIPTION: Twelve months, 6/6; Six months, 4/-; Three months, 2/-, post free to any address in the United Kingdom. Abroad, Twelve months, 8/8; Six months, 5/-; Three months, 2/6, post free.—Address The Subscription Dept., "THE HOSPITAL," The Hospital Building, 28/29 Southampton Street, Strand, London, W.C.

